# Quantitative Assessment of Tumor Contact with Neurogenic Zones and Its Effects on Survival: Insights beyond Traditional Predictors

**DOI:** 10.3390/cancers16091743

**Published:** 2024-04-29

**Authors:** Kirsten Jung, Johanna Kempter, Georg Prokop, Tim Herrmann, Michael Griessmair, Su-Hwan Kim, Claire Delbridge, Bernhard Meyer, Denise Bernhardt, Stephanie E. Combs, Claus Zimmer, Benedikt Wiestler, Friederike Schmidt-Graf, Marie-Christin Metz

**Affiliations:** 1 Department of Neuroradiology, School of Medicine and Health, Technical University of Munich, 81675 München, Germany; tim.herrmann@tum.de (T.H.); michael.griessmair@tum.de (M.G.); suhwan.kim@tum.de (S.-H.K.); claus.zimmer@tum.de (C.Z.); b.wiestler@tum.de (B.W.); marie.metz@tum.de (M.-C.M.); 2 Department of Neurology, School of Medicine and Health, Technical University of Munich, 81675 München, Germany; johanna.m.kempter@gmail.com (J.K.); georg.prokop@tum.de (G.P.); f.schmidt-graf@tum.de (F.S.-G.); 3 Department of Pathology, School of Medicine and Health, Technical University of Munich, 81675 München, Germany; c.delbridge@tum.de; 4 Department of Neurosurgery, School of Medicine and Health, Technical University of Munich, 81675 München, Germany; 5 Department of Radiation Oncology, School of Medicine and Health, Technical University of Munich, 81675 München, Germany; denise.bernhardt@tum.de (D.B.); stephanie.combs@tum.de (S.E.C.); 6 TranslaTUM, Technical University of Munich, 81675 München, Germany

**Keywords:** glioblastoma, computational, quantitative, SVZ, SGZ, survival

## Abstract

**Simple Summary:**

The exact cellular origin of glioblastoma (GBM) remains uncertain, with prevalent theories suggesting emergence from transformed endogenous stem cells. These cells likely play pivotal roles in tumor initiation and progression. The impact of proximity of GBM to the brain’s neurogenic zones on patient survival remains a lingering question. Our study investigated tumor infiltration into the subventricular zone (SVZ), the subgranular zone (SGZ) and the cortex alongside clinical variables, such as KPS score, multifocality and selected molecular markers. Utilizing a fully automated processing and segmentation pipeline, we objectively quantified these relationships in 177 IDH wild-type glioblastomas. Our findings support prior research indicating SVZ proximity as a predictor for poor survival, while SGZ proximity showed no significant impact. We also established new survival thresholds based on tumor mass fractions and minimal distances. Contrary to previous studies, we found no significant correlations between SVZ contact and multifocal growth pattern or MGMT promoter methylation.

**Abstract:**

So far, the cellular origin of glioblastoma (GBM) needs to be determined, with prevalent theories suggesting emergence from transformed endogenous stem cells. Adult neurogenesis primarily occurs in two brain regions: the subventricular zone (SVZ) and the subgranular zone (SGZ) of the hippocampal dentate gyrus. Whether the proximity of GBM to these neurogenic niches affects patient outcome remains uncertain. Previous studies often rely on subjective assessments, limiting the reliability of those results. In this study, we assessed the impact of GBM’s relationship with the cortex, SVZ and SGZ on clinical variables using fully automated segmentation methods. In 177 glioblastoma patients, we calculated optimal cutpoints of minimal distances to the SVZ and SGZ to distinguish poor from favorable survival. The impact of tumor contact with neurogenic zones on clinical parameters, such as overall survival, multifocality, MGMT promotor methylation, Ki-67 and KPS score was also examined by multivariable regression analysis, chi-square test and Mann–Whitney-U. The analysis confirmed shorter survival in tumors contacting the SVZ with an optimal cutpoint of 14 mm distance to the SVZ, separating poor from more favorable survival. In contrast, tumor contact with the SGZ did not negatively affect survival. We did not find significant correlations with multifocality or MGMT promotor methylation in tumors contacting the SVZ, as previous studies discussed. These findings suggest that the spatial relationship between GBM and neurogenic niches needs to be assessed differently. Objective measurements disprove prior assumptions, warranting further research on this topic.

## 1. Introduction

Glioblastomas (GBMs), isocitrate dehydrogenase 1 or 2 (IDH) wildtype, represent the most aggressive and malignant form of primary brain tumors. They are characterized by their infiltrative growth and resistance to conventional therapeutic interventions, resulting in a median overall survival in large epidemiological studies of real live data of 9–12 months [1,2].

One of the current challenges is understanding the intratumoral heterogeneity of GBMs, which is crucial for addressing treatment resistance and relapse. The precise cellular origin of glioblastoma (GBM) remains to be conclusively determined. However, various cell types within the central nervous system (CNS), such as neural precursor cells (NPC), oligodendrocyte precursor cells (OPC), and astrocytic precursor cells (APC), have been proposed as potential contenders for initiating GBM [3,4]. It is also suggested that glioma ontogeny is connected to a subpopulation of neural stem (NSC) and NPC found in NSC-like astrocytes. Adult neurogenesis, the process of generating new neurons in the adult brain, is limited and active in two main areas: the subgranular zone (SGZ) of the dentate gyrus (DG) in the hippocampus and the subventricular zone (SVZ) [5,6,7,8,9]. The SVZ is found just under the ependyma of the lateral brain ventricles and consists of four layers (layers I–IV) of which astrocyte-like NSCs and neuroblasts can be found in layer III [7,10]. It is where stem cells reside and undergo differentiation into neurons and glial cells during the later stages of embryonic development [11].

The search for predictive parameters to optimize patients’ treatment has been a longstanding research objective. Long-term survival in patients with GBM is uncommon, with fewer than 7% of patients surviving beyond five years, and a significant portion of these individuals experience irreversible neurological deficits [12]. Typically, factors linked to extended survival encompass younger age, favorable Karnofsky performance status (KPS) at the time of diagnosis, and the presence of O6-Methylguanine-DNA methyltransferase (MGMT) promoter methylation as a predictive marker of benefit from alkylating agents like temozolomide—and as strong prognostic marker as well [1,13,14]. Age impacts treatment decisions, with younger patients often opting for debulking surgery over biopsy and having more therapy options, leading to prolonged survival outcomes [13]. Generally, the presence of an IDH mutation, more common in younger patients, also correlates with extended survival. The presence of an IDH mutation represents an initial occurrence in tumorigenesis, leading to tumors that are clinically and genetically different from those lacking the IDH mutation. This led to a distinct entity in the updated 2021 WHO classification, termed “astrocytoma, IDH-mutant, CNS WHO grade 4” [13,15]. Thus, IDH mutation technically does not affect survival of correctly defined GBM patients anymore.

Further, tumors located in the temporal, occipital, and parietal lobes are associated with improved survival rates [16,17]). Conversely, areas associated with lower survival rates include central areas, such as basal ganglia and corpus callosum [16,17].

In general, contrast enhancement reaching the cortex was independently associated with longer overall survival in a large retrospective study of 1006 glioblastoma patients [18]. Interestingly, in the same study, tumor-related epilepsy at diagnosis also was a predictor of a more favorable survival independent of tumor location and volume, age, KPS score, extent of resection, radiochemotherapy, levetiracetam use and MGMT promotor methylation.

On a similar note, multiple studies reported that cortex involvement by the tumor predicts tumor-related epilepsy, suggesting that this symptom might be triggered by interaction between glioblastoma cells and the functional neocortex [18,19,20,21]. However, the complex interaction of cortex involvement, epileptic seizures and prognosis of glioblastoma is scarcely investigated so far.

According to other studies, the infiltration of glioblastoma in the SVZ emerges as an independent prognostic factor for unfavorable survival [5,22,23,24,25]. Tumors in contact with the SVZ seem to be linked to more aggressive recurrences and a higher frequency of contralateral relapses [26]. Due to the theory of GBM growth being connected to the SVZ, studies took a closer look at the MRI features of GBMs in specific relation to the SVZ. Lim et al. employed a technique where they manually determined the relationship of the contrast-enhancing tumor (CET) with the SVZ and cortex preoperatively. In order to predict tumor recurrence, they subjectively classified the tumors into four groups: CET touching the SVZ and infiltrating the cortex (group I), CET contacting the SVZ but not involving the cortex (group II), CET not contacting the SVZ but involving the cortex (group III) and CET neither contacting the SVZ nor infiltrating the cortex (group IV). In their cohort of 53 patients, they found that glioblastoma growing inside neural stem cell areas appeared to be multifocal more often and had recurrences farther away from the initial lesion [10]. Tumors without contact with either the cortex or the SVZ never showed a multifocal growth pattern.

Our paper takes an approach to investigate the effects of tumor contact with the SVZ, the SGZ or the cortex in a fully automated, quantitative classification based on established atlases rather than using qualitative measurements as done before. We aim to contribute to the ongoing discourse on glioblastomas, providing insights that may guide a better prediction on survival and location of relapses.

## 2. Materials and Methods

### 2.1. Patient Selection and Patient Data

In this retrospective study, we examined a subset of our institutional observational cohort of adult glioma patients (n = 177) diagnosed with a GBM, IDH wildtype as per the WHO 2021 classification of tumors of the CNS. These patients were treated for newly diagnosed GBM at the local department of neurosurgery between 2014 and 2017. Inclusion criteria were availability of demographic, treatment-related and survival outcome data, neuropathological and molecular assessments including IDH status, MGMT promotor methylation status and Ki-67 proliferation index, as well as preoperative MRI, which was accessible on the local picture archiving and communication system (PACS). Minimum MRI protocol requirements were 3D T1 before and after gadolinium-based contrast administration, 2D or 3D fluid-attenuated inversion recovery (FLAIR) and/or conventional T2 scan. In adherence to the Declaration of Helsinki and the approved research protocols by the Institutional Review Board, informed consent was acquired from all participants (Ethical code: #340/16S). Histological diagnosis was validated through state-of-the-art neuropathological assessment. The IDH1 mutation and MGMT promoter methylation status, as well as the percentage of Ki-67 expression, were determined following established protocols. Adjuvant treatments included radiotherapy, concurrent and/or standalone TMZ-based chemotherapy and participation in clinical trials. Tumor progression was defined according to the Response Assessment in Neuro-Oncology (RANO 1.0) criteria, with salvage therapies determined by interdisciplinary consensus. Salvage treatments encompassed various approaches such as re-resection, re-irradiation and cytotoxic agents. OS was calculated from the date of initial resection to death.

### 2.2. Image Acquisition and Post-Processing

Preoperative MR imaging was primarily performed using a Philips 3 Tesla whole-body scanner (Achieva or Ingenia, Philips, Best, The Netherlands) or a Siemens Verio 3 Tesla whole-body scanner (Siemens, Erlangen, Germany). As stated above, imaging protocols at least included an isotropic T1-weighted turbo field echo (TFE) sequence with a voxel size of 1 mm^3^ acquired both pre- and post-contrast administration, isotropic FLAIR with a voxel size of 1 mm^3^ and/or an axial T2-weighted sequence with a voxel resolution of 0.36 × 0.36 × 4 mm^3^. All images were rigidly co-registered into the SRI24 atlas space using NiftyReg and skull-stripped using HD-BET [27,28]. Next, we automatically segmented tumor subregions into contrast-enhancing tumor (CET), necrosis and edema by applying the freely available BraTS Toolkit, which assembles various state-of-the-art image segmentation algorithms [29]. In case of missing T2-weighted or FLAIR images, those sequences were automatically synthesized using a GAN-based approach to improve automated segmentation [30]. This generative adversarial network was previously trained in a many-to-many mapping strategy and enables the robust synthesis of missing sequences (e.g., T2w) from available input sequences. Its positive impact on segmentation performance has been validated in [30]. All registration and segmentation post-processing outcomes underwent visual confirmation by Kirsten Jung, Michael Griessmair and Marie-Christin Metz.

### 2.3. Automated Segmentation of Neurogenic Zones

To automatically segment neurogenic zones in the preoperative MRI, we followed the methodology developed by [31]. In brief, we first employed ANTs (using the parameters established as a strong baseline for the BraTSReg challenge [32] to deformably register the SRI atlas onto the patient’s preoperative MR image and then warped the SVZ atlas (from Bruil et al. [31]) and the Julich brain atlas for segmentation of the DG [33] into patient anatomy. All registrations were visually quality checked.

**Figure 1:**
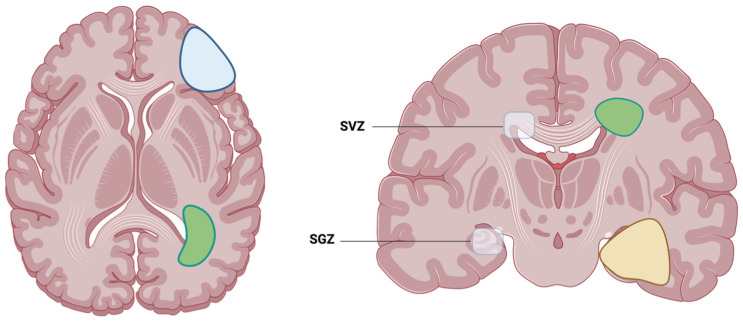
Illustration of the two locations where adult neurogenesis is located: SVZ, which is the subventricular zone, and SGZ, which is the subgranular zone. Examples of possible glioblastoma contact with either the cortex (blue), SVZ (green) or SGZ (yellow). Created with BioRender.com (accessed on 22 March 2024).

**Figure 2:**
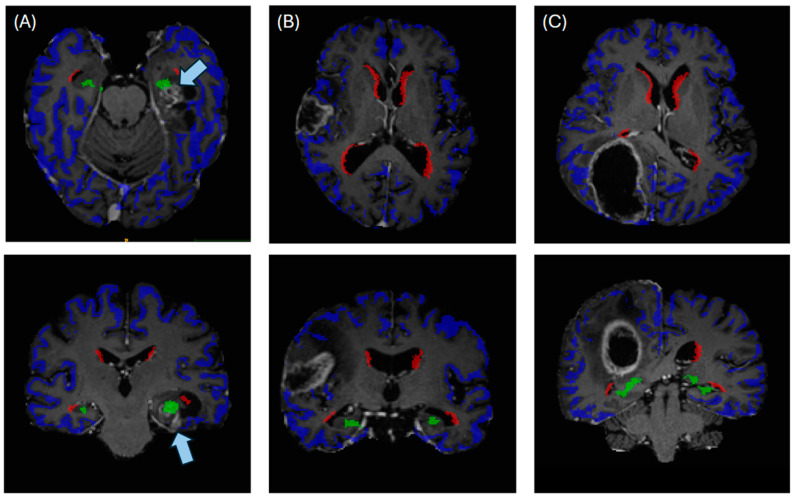
Illustrating the automated anatomical segmentations for cases of (**A**) a tumor contacting subgranular zone (green) and cortex (blue), (**B**) a tumor contacting the cortex and (**C**) a tumor contacting cortex and subventricular zone (red). Arrows in (**A**) indicate the localization of the contrast-enhancing tumor.

Following this automated tumor and atlas segmentation, we decided to analyze the overall survival and other clinical factors (as described in Section 2.4) of GBM with either contact with the cortex, the SVZ or the SGZ, as illustrated in Figure 1 and Figure 2. For each tumor, its center of mass (of tumor core, i.e., contrast-enhancing tumor and necrosis) was calculated (using SciPy) and the minimum Euclidean distance to the respective neurogenic zones was automatically determined using SciPy distribution functions.

### 2.4. Statistical Analysis

Descriptive statistics, including means, standard deviation (SD) and frequencies (percentages), were used to describe patient characteristics (age, gender, primary tumor site, resection status, KPS at diagnosis and preoperative seizures) and survival status. All statistical analyses were carried out in Python 3.9, utilizing the open-source libraries scikit-learn 1.2.2, scikit-image 0.21, SciPy 1.12.0, and lifelines version 0.28.0, or in R (version 4.3), using the maxstat package (version 0.7–25). We performed two main sets of analyses. First, we performed various survival analyses to examine the effect of tumor contact with the SVZ, SGZ and cortex on patients’ overall survival. Here, we included established predictive factors, namely tumor volume, patient age at diagnosis, tumor resection, MGMT promotor methylation status, KPS score and multifocality at initial diagnosis, as well as the quantitative markers of tumor contact with neurogenic zones, i.e., minimal distance to the SVZ and SGZ, respectively, and tumor fraction in the SVZ, SGZ and cortex. After checking for collinearity of the variables, we constructed multivariable regression models using Cox proportional hazard models with OS. To further look into our quantitative data, we calculated optimal cutpoints of relative tumor portions in the SVZ, SGZ and cortex and minimal distances to the SVZ and SGZ to distinguish poor from favorable survival using maximally selected rank statistics with MC-based *p*-value adjustment in R. Secondly, we examined the relationship between contact with neurogenic zones/cortex and selected clinical parameters, namely overall survival, multifocality (during the entire course of disease), MGMT promotor methylation, Ki-67 proliferation index (defined as >30%, <30% or not evaluated), numeric KPS score and a history of epileptic seizures at initial diagnosis. Tumor contact was included by binarizing SVZ, SGZ and cortex contact from the respective quantitative percentages of tumor fractions. To evaluate significant differences between groups, one-way chi-square test, paired t-test as well as a non-parametric Mann–Whitney-U test were performed with a significance level of 0.05.

In the case of a multifocal tumor appearance preoperatively, the tumor subportion with the greatest automatically defined tumor volume was evaluated for the clinical correlation analyses. This decision was driven by visual inspection of those tumors that mostly showed one main tumor portion and between one and four small, partially tiny tumor lesions.

## 3. Results

### 3.1. Characteristic of the Study Population

Demographics included age at initial diagnosis and sex. Clinical characteristics included the overall survival, calculated as the difference between the first resection and the date of death. Other clinical characteristics included the extent of resection (gross total (GTR), subtotal resection (STR) and biopsy), tumor location (frontal, parietal, temporal, occipital, central, and/or other or multifocal), Karnofsky performance status and preoperative epilepsy, MGMT promoter methylation status and Ki-67 status. Participant demographics and clinical characteristics are listed in Table 1.

### 3.2. Results of Survival Analysis with Quantitative Localization Data

Since the correlation matrix logically showed high collinearity for distance to the SVZ/SGZ and respective tumor percentages in these areas (see Supplementary Figure S1), we included them in two separate multivariable regression models. Other collinearities, such as distance to the SVZ and distance to the SGZ were deliberately accepted. Results of the Cox proportional hazard models can be found in Table 2. In both models, the known risk factor patient age at diagnosis was significantly associated with poor survival, while tumor resection (which in this case included subtotal and gross tumor resection) and higher KPS score at initial diagnosis were significant predictors for a favorable survival (*p* < 0.05). Under these conditions, increasing distance to the SVZ also was a significant predictor for prolonged survival (*p* = 0.01). In contrast, increasing distance to the SGZ was associated with poor survival, although not on a significant level (*p* = 0.08). The same held true for the relative tumor fraction in the cortex (*p* = 0.87) and relative tumor fraction in the SGZ (*p* = 0.92). Preoperative tumor volume showed no correlation with survival (*p* < 0.005).

**Figure 3:**
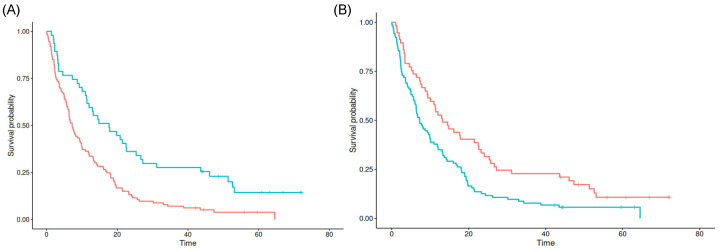
Kaplan–Meier survival estimates for SVZ contact (**A**) and SVZ distance (**B**). Blue lines denote tumors with values above the cutpoint, red lines for tumors with values below the cutpoint.

Interestingly, the cutpoint of 14.07 mm distance to the SVZ and a relative tumor fraction of 0.03% in the SVZ seem to best separate poor from favorable prognosis in the maxstat analysis (*p* = 0.027 and *p* < 0.001), respectively. For both the minimum distance to the SGZ as well as relative tumor fractions in the SGZ and cortex, no significant cutpoints could be found (see Figure 3).

### 3.3. Correlations between Localization Data and Clinical Parameters

All 177 tumors were separated into subgroups with (n = 128) and without SVZ contact (n = 49), based on the objective segmentations as described in the Methods Section. Tumor contact was defined by a relative fraction of >0% in the SVZ. Similarly, n = 54 were grouped as tumors with contact with the SGZ (while n = 123 showed no contact with the SGZ), and n = 160 tumors had contact with the cortex, while a remarkably small group of n = 17 tumors had no contact with the cortex, based on automated segmentations. For all location-dependent subgroups, Table 3 lists overall survival, prevalence of preoperative or general multifocality of the tumors, absolute and relative numbers of MGMT promotor methylation status, Ki-67 proliferation index (defined as above or below 30%, or not evaluated), median and mean KPS score and prevalence of epileptic seizures at the time of initial diagnosis. Median overall survival was significantly longer in tumors without SVZ contact (6.97 vs. 12.49 months, *p* = 0.008). There were no significant differences in survival regarding SGZ or cortex contact or not.

In contrast to prior studies (as elaborated later in the Discussion Section), we found no significant differences between tumors with and without SVZ contact looking at the frequencies of MGMT promotor methylation status or multifocal tumor growth. Nevertheless, with a lower percentage of MGMT promotor methylated tumors in the subgroup with SVZ contact (37.5% vs. 42.8%, *p* = 0.666), it tended towards the previously described observations.

**Figure 4:**
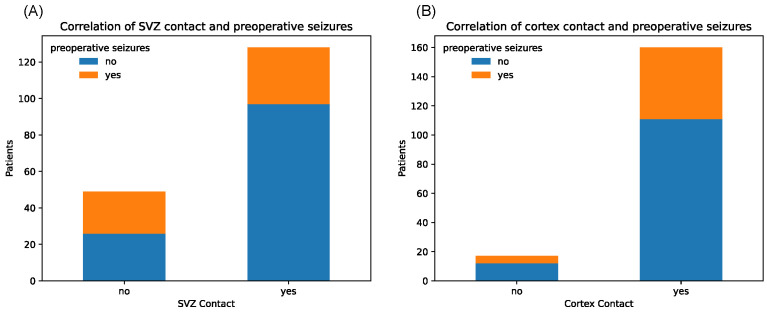
Counts of patients with and without history of preoperative seizures, depending on binary tumor contact with SVZ (**A**) and cortex (**B**), respectively.

Notably, tumors with SVZ contact caused significantly fewer epileptic seizures in the preoperative setting (*p* = 0.006, chi-square test) (Figure 4).

**Figure 5:**
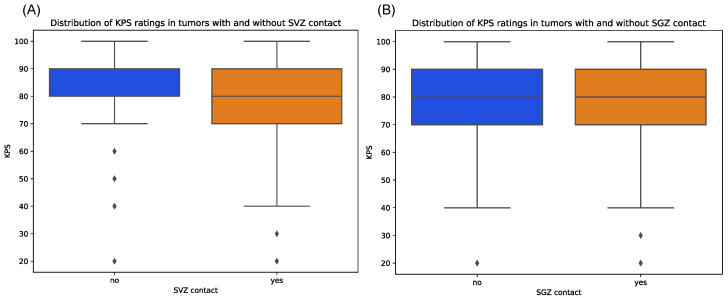
Distribution of preoperative KPS scores for patients with tumors contacting SVZ (**A**) and SGZ (**B**), respectively, compared to those contacting neither region. Diamonds denote outliers.

Contrarily, tumors with cortex contact were more often associated with preoperative seizures, although not on a significant level, which might be due to the unequal sample sizes (*p* = 1.00). Median KPS score at initial diagnosis showed relatively equal distributions between tumors with and without SGZ and cortex contact but were slightly lower in the group with SVZ contact with a median of 80 vs. 90 in the non-SVZ-contact group (*p* = 0.666) (Figure 5). Further, there were no significant differences in Ki-67 proliferation indices between all groups.

## 4. Discussion

The exact role of neurogenic zones in the development of malignant brain tumors is yet to be determined [22,34,35]. Several studies have shown an association of glioblastoma involvement in the SVZ, a neurogenic niche located along the lateral wall of the ventricles, with lower survival rates [4,22,23,25]. In contrast, fewer studies have assessed the effects of tumor involvement of the SGZ, another neurogenic zone in the dentate gyrus of the hippocampus [31,36]. While this did not seem to affect the survival of glioblastoma patients in a study by Mistry and colleagues ([31,36], irradiation of the SGZ can result in cognitive decline, proving its sensitivity to local disturbances [37].

In this study, we investigated the impact of SVZ, SGZ and cortex involvement of 177 IDH wildtype glioblastomas on survival, as well as their relation to other clinical variables, such as KPS score, epileptic seizures at initial diagnosis, multifocality and selected molecular markers (MGMT promotor methylation status and Ki-67 proliferation index).

Most of the prior studies addressing SVZ and SGZ involvement of brain tumors were based on subjective readings by individuals, potentially introducing error and hindering reproducibility [5,10,23]. In a recent study, Bruil et al. developed an SVZ atlas to facilitate reproducible research on this topic [31]. While they focused their work on the impact of SVZ and SGZ irradiation on survival of glioma patients, studies that examine the interaction of SVZ/SGZ involvement of brain tumors and clinical cofactors based on these objective definitions are currently lacking. Therefore, we gratefully utilized their SVZ atlas and the also openly available Julich brain atlas [33] for definition of the dentate gyrus as a proxy of the SGZ, aiming to validate or revise prior observations on clinical implications of SVZ/SGZ contact by—for the first time—using completely objective and reproducible quantitative measurements. In accordance with similar studies, we also assessed the effects of cortex involvement of the tumors, which in turn was objectively defined by utilizing automatically generated tumor segmentations and tissue maps.

Our results confirm the prior findings of Hallaert et al. and Flores et al., who also could not find a correlation between preoperative tumor volume and survival [38,39]. We found significantly shorter overall survival times in tumors contacting the SVZ in comparison to distant ones [5,22,23,24,25]. In our cohort, median survival was 6.97 months for the SVZ-contacting group (n = 128) in contrast to 12.49 months for the group without SVZ contact (*p* = 0.008). The multivariable regression analysis also identified the minimal distance to the SVZ as an independent predictor for survival (HR: 0.97, *p* = 0.01), which translates into a worse survival the closer the tumor is to the SVZ. This held true even when established risk factors, such as patient age at diagnosis, KPS score and resection, were included in the model. In fact, patients with tumors contacting the SVZ also exhibited lower KPS scores at initial diagnosis (with a median KPS score of 80% vs. 90% in the non-contact group), although this difference was not statistically significant (*p* = 0.666).

Further, we aimed to determine the exact distance to the SVZ that will make a noticeable difference in survival outcome, utilizing our objective quantitative measurements. A prior study did not find a correlation between overall survival and the (in their case manually drawn) shortest distance to the SVZ [40]. In contrast to them, we defined the minimal distance as the shortest distance from the center of mass to the SVZ delineation (and not from the enhancing edge) and calculated it automatically. With this method, we found a distance of 14.07 mm to be the optimal cutpoint distance to differentiate poor from favorable survival, as determined by maxstat analysis (*p* = 0.027). Depending on the volume of the contrast-enhancing tumor portion, this might or might not include non-enhancing, edematous tumor parts as well. Further studies are needed to investigate the impact of SVZ involvement by non-enhancing tumor areas specifically.

In terms of relative tumor fractions involving the SVZ, a portion of 0.03% of the whole tumor volume is enough to determine a significantly worse survival as opposed to tumors with fewer involvement fractions, as our maxstat analysis revealed (*p* < 0.001), essentially saying that virtually any form of SVZ contact distinguishes a group of GBM patients with shorter survival.

This might explain why the relative tumor fraction in the SVZ turned out not to be an independent risk factor for survival in our multivariable regression model, in contrast to distance to the SVZ. Apparently, marginal SVZ contact is sufficient to influence survival, while there is no linear relationship of an increasingly worse survival rate with gradually more SVZ involvement.

Interestingly, in our study, tumor proximity to the SGZ seems to be a weak predictor for a more favorable survival as minimal distance to SGZ in the multivariable regression model showed a hazard ratio of 1.01 (*p* = 0.08) and relative tumor fraction in SGZ had a hazard ratio of 0.51 (*p* = 0.92). Of note, these were only tendencies that did not show statistical significance. In fact, median survival times for the SGZ-contacting and non-contacting groups were quite similar (7.28 and 9.25 months). However, even if SGZ contact is interpreted as completely irrelevant for survival prediction, it stands in clear contrast to the other neurogenic zone, i.e., the SVZ. This observation is remarkable since the density of proliferative cells and their age-related alterations is similar in both the SVZ and SGZ [41]. Making similar observations, Mistry and colleagues hypothesized that extensive contact of the SVZ with cerebrospinal fluid as well as the presence of a fine, hypocellular gap layer filled with astrocytic processes in the SVZ contribute to an environment that supports tumor growth, e.g., by growth factors enriched in the cerebrospinal fluid [36,42]. The observation of ependymal tumor dissemination through cerebrospinal fluid or along the length of the SVZ also supports this hypothesis [43].

In contrast to the study by Mistry and colleagues, who noted a significantly lower median KPS in patients with GBMs contacting the SGZ, we did not find significant differences as in our cohort median KPS score was 80% for both groups. However, since the SGZ generates neural stem cells which are involved in pathways of memory and learning, one might expect a slightly worse operational performance among patients having tumors in this neurogenic zone [44]. Also, as stated above, irradiation of the SGZ can result in cognitive decline [37]. In general, KPS scores are based on the judgment of individual healthcare professionals, which can introduce subjectivity and variability in scoring. Therefore, such results need to be carefully interpreted.

In contrast to prior studies, we could not reproduce a significant correlation between SVZ contact and multifocal tumor growth, neither in the preoperative stage nor during tumor progression [10,45]. Multifocality is one frequently and controversially discussed explanation for the worse survival outcome of SVZ-contacting glioblastomas. Another one is the hypothesis that SVZ-contacting tumors harbor a higher expression of MGMT and a lower rate of MGMT promotor methylation. MGMT encodes a DNA repair protein which is known to restore temozolomide damage [46]. Interestingly, maintenance of the glioblastoma stem cell state has been found to correlate with MGMT expression [47].

Steed et al. found that glioblastomas with a centroid located in proximity to the SVZ (with a rather arbitrary maximum distance of 19.23 mm) exhibited higher expression of MGMT [35]. While we did not test for MGMT expression levels in our study, we examined the previously described correlation of SVZ contact and MGMT promotor methylation status in glioblastomas [25]. This epigenetic modification of the cytosine–phosphate–guanine (CpG) island within the promotor region of the MGMT gene is known to silence this gene, leading to enhanced response to temozolomide [48]. Unlike Zhao et al., who discussed a lower rate of MGMT promotor methylation as a possible reason for the poorer prognosis of SVZ-involving gliomas, we did not find significant differences in MGMT promotor methylation between SVZ-contacting and non-contacting tumors. Therefore, we encourage further research on the complex interaction of SVZ involvement, MGMT expression and MGMT promotor hypermethylation.

In general, the complex interaction of specific genetic alterations in SVZ-contacting tumors with clinicoradiological phenotype is an exciting question for further studies [49].

Interestingly, in our cohort, patients with glioblastomas contacting the SVZ significantly rarer had a history of epileptic seizures in comparison to those with tumors outside the SVZ (*p* = 0.006). This might be due to a greater distance to the cortex (which usually is associated with a higher incidence of epileptic seizures) or structural differences in the local tumoral microenvironment that prevent epileptogenesis, such as a less pronounced decrease in inhibitory GABA-ergic neurotransmission, which is known to promote seizures in glioma patients [18,50]. With those observations, we encourage further experimental research on this interesting topic.

Also, the relation of SVZ/SGZ involvement and other typical symptoms GBM patients suffer from, such as headaches or nausea, might be addressed in future studies. However, the lack of objectivity of those symptoms has to be taken carefully into consideration.

Lastly, we did not see differences in Ki-67 proliferation indices between SVZ/SGZ-contacting tumors and those without contact with the neurogenic zones. While this topic is scarcely investigated so far, in general, studies found that in the SVZ the proliferative marker Ki-67 decreases during aging, and in the adult SVZ, Ki-67 is exclusively expressed by microglia, the primary resident immune cells of the brain [51].

### Limitations of the Study

While this study offers valuable insights, it is important to acknowledge various limitations that warrant careful consideration when interpreting the findings.

Initially, it is essential to acknowledge that the study was conducted retrospectively at a single center, raising potential concerns regarding the broader applicability of our results. Nevertheless, it is noteworthy that our cohort encompassed a relatively sizable number of patients, which could partially offset these constraints.

One potential limitation could be that the segmentation of the SGZ, SVZ and cortex was conducted automatically, which could harbor some degree of systemic bias, although we visually checked the segmentations for plausibility.

As described in the Methods Section, in case of a multifocal tumor in the preoperative setting, after automated image processing and segmentation, the tumor portion with the largest volume was selected for further analysis, i.e., for definition of SVZ/SGZ/cortex contact in the correlation analyses with clinical markers and in the multivariable Cox regression models. Although we decided that this was the most appropriate solution in order to investigate those interactions on a patient-based (instead of a tumor focus-based level), this might introduce some bias in terms of tumor volume and interpretation of MGMT promotor methylation and Ki-67 proliferation indices, respectively (since those molecular data might come from sampling of another tumor lesion). However, by visual inspection of our cases, we noted that most of the patients had one large tumor focus, and one–three tiny satellite lesions, which should not influence the total tumor volume significantly.

In terms of the multivariable regression analysis, we had to decide on a limited set of the most important established predictors in order to still obtain interpretable results considering our small sample size of 177 patients.

Further, this is a censored model, in which 12 patients were included that were still alive at the time of censoring.

Lastly, in the correlation analyses, we only investigated the effects of binary contact with the SVZ, SGZ and cortex separately and did not look specifically into the “overlapping” situations of tumors, for example infiltrating the SVZ and SGZ concurrently.

## 5. Conclusions

The various interactions of glioblastomas contacting neurogenic zones, such as the SVZ and SGZ, and their clinical geno- and phenotype have been controversially discussed over the last decade. Aiming to enhance the reproducibility and objectiveness of this topic, we applied a fully automated post-processing and segmentation pipeline for the definitions of tumor contact with the SVZ, SGZ and cortex by utilizing established atlases. While our results confirmed prior studies by identifying proximity to the SVZ as an independent predictor of poor survival, we further investigated this correlation and calculated the optimal cutpoint in minimal distance to the SVZ that separates poor survival from a more favorable one. In contrast to that, proximity or infiltration of the SGZ did not significantly affect survival times. Other controversial hypotheses, such as the correlation of SVZ involvement and MGMT promotor methylation or multifocality, could not be supported by our results.

## Figures and Tables

**Table 1:** Characteristics of the study population.

Patients Characteristics	Total (n = 177)
Age at time of initial diagnosis (mean)	
male	63.99 years
female	62.68 years
all	65.51 years
Gender (absolute and in percent)	
male	95 (53.67%)
female	82 (46.33%)
Preoperative seizures (absolute and in percent)	54 (30.51%)
KPS at diagnosis (median)	80
Resection status (absolute and in percent)	
biopsy	69 (38.98%)
resection (subtotal or gross total)	107 (60.45%)
unknown	1 (0.56 %)
Tumor location (absolute and in percent)	
frontal	34 (19.21%)
central	20 (11.30%)
temporal	59 (33.33%)
parietal	28 (15.82%)
occipital	8 (4.52%)
multifocal	20 (11.30%)
others	8 (4.52%)
Overall survival (median)	8.10 months
PFS (median)	3.97 months

**Table 2:** Results of the multivariable regression analysis. The upper part shows results of the first multivariable regression analysis, including minimal distance to SVZ and SGZ. The lower part presents results of the second analysis, including relative tumor fraction in SVZ, SGZ and cortex. *p* values below the significance level of 0.05 are highlighted in bold.

Variable	HR (95% CI)	*p* Value
Tumor volume (main component) in mm^3^	1.00 (1.00–1.00)	**<0.005**
Patient age at diagnosis	1.06 (1.04–1.07)	**<0.005**
Resection (at any time)	0.26 (0.17–0.41)	**<0.005**
MGMT Promotor Methylation	1.01 (1.00–1.02)	0.18
KPS score	0.99 (0.99–1.00)	**0.01**
Multifocality at initial diagnosis	1.67 (0.98–2.86)	0.06
**Relative tumor fraction in SVZ in %**	117.77 (0.08–1.80 × 10^5^)	0.20
**Relative tumor fraction in SGZ in %**	0.51 (0.00–1.46 × 10^5^)	0.92
**Relative tumor fraction in cortex in %**	0.26 (0.17–0.41)	0.87
**_________________________________________**		
Tumor volume (main component) in mm^3^	1.00 (1.00–1.00)	**<0.005**
Patient age at diagnosis	1.06 (1.04–1.07)	**<0.005**
Resection (at any time)	0.24 (0.16–0.36)	**<0.005**
MGMT promotor methylation	1.01 (1.00–1.02)	0.21
KPS score	0.99 (0.99–1.00)	**0.01**
Multifocality at initial diagnosis	1.51 (0.89–2.57)	0.12
**Minimal distance to SVZ in mm**	0.97 (0.95–0.99)	**0.01**
**Minimal distance to SGZ in mm**	1.01 (1.00–1.02)	0.08
**Variable**	**HR (95% CI)**	***p* value**

**Table 3:** Correlations between localization data and clinical parameters grouped as tumors with contact either with subventricular zone (SVZ), subgranular zone (SGZ) or cortex. P values below the significance level of 0.05 are highlighted in bold.

		Tumor Contact with SVZ			Tumor Contact with SGZ			Tumor Contact with Cortex		
Variables		SVZ Contact	No SVZ Contact	*p* Value	SGZ Contact	No SGZ Contact	*p* Value	Cortex Contact	No Cortex Contact	*p* Value
	Total Counts	128 (72.3%)	49 (27.7%)		54 (30.5%)	123(69.5%)		160 (90.4%)	17 (9.6%)	
**Overall survival**	Median	6.97	12.49	**0.008**	7.28	9.25	0.690	8.10	10.20	0.824
**Multifocality**	**Preoperative**									
	Yes	13 (10.2%)	7 (14.3%)	0.609	2 (3.7%)	18 (14.6%)	0.063	18 (11.2%)	2 (11.8%)	1.00
	No	115 (89.8%)	42 (85.7%)		52 (96.3%)	105(85.4%)		142 (88.8%)	15 (88.2%)	
	**In total**									
	Yes	40 (31.2%)	19 (38.8%)	0.440	14 (25.9%)	45 (36.6%)	0.225	52 (32.5%)	7 (41.1%)	0.652
	No	88 (68.8%)	30 (61.2%)		40 (74.0%)	78 (63.4%)		108 (67.5%)	10 (58.8%)	
**MGMT promotor methylation**	Yes	48 (37.5%)	21 (42.8%)		17 (31.5%)	52 (42.3%)		63 (39.4%)	6 (35.3%)	
	No	76 (59.4%)	27 (55.1%)	0.666	35 (64.8%)	68 (55.3%)	0.255	92 (57.5%)	11 (64.7%)	0.868
	Not evaluated	4 (3.1%)	1 (2.0%)		2 (3.7%)	3 (2.4%)		5 (3.1%)	0 (0%)	
**Ki-67 Proliferation index**	>30%	49 (38.3%)	22 (44.9%)		22 (40.7%)	49 (39.9%)		64 (40.0%)	7 (41.1%)	
	<30%	37 (28.9%)	19 (38.7%)	0.872	15 (27.8%)	41 (33.3%)	0.749	48 (30.0%)	8 (47.1%)	0.624
	Not evaluated	42 (32.8%)	8 (16.3%)		17 (31.5%)	33 (26.8%)		48 (30.0%)	2 (11.8%)	
**KPS score**	Median	80	90	0.666	80	80	0.749	80	80	
**Epilepsy**	Yes	31 (24.2%)	23 (46.9%)		17 (31.5%)	37 (30.1%)		49 (30.6%)	5 (29.4%)	
	No	97 (75.8%)	26 (53.1%)	**0.006**	37 (68.5%)	86 (69.9%)	0.993	111 (69.4%)	12 (70.6%)	1.00

## Data Availability

The clinical and imaging data are not publicly available due to privacy restrictions.

## Supplementary Materials

The following supporting information can be downloaded at https://www.mdpi.com/article/10.3390/cancers16091743/s1, Figure S1: Correlation matrix indicating high or low correlation between clinical and/or localization parameters in a color-coded heatmap. Within this matrix, each cell signifies the Pearson’s correlation coefficient between two specific variables.

## Abbreviations

CET	Contrast-enhancing tumor
CNS	central nervous system
GBM	Glioblastoma
WHO	World Health Organization
TMZ	temozolomide
OPC	oligodendrocyte precursor cells
APC	astrocytic precursor cells
NSC	neural stem cell
NPC	neural progenitor cell
SVZ	subventricular zone
PFS	progression-free survival
OS	overall survival
IDH1	isocitrate dehydrogenase
KPS	Karnofsky performance status
MGMT	O6-methylguanin-DNA-methyltransferase
CE	contrast-enhancing
TFE	T1-weighted Turbo Field Echo
FLAIR	fluid-attenuated inversion recovery
RT	radiotherapy
HR	hazard ratio
CI	confidence intervall
PACS	Picture Archiving and Communication System
RANO	Response Assessment in Neuro-Oncology
MPRAGE	magnetization-prepared rapid gradient-echo
GTR	gross total resection
STR	subtotal resection
SD	Standard Deviation
DG	Dental gyrus
