# Supplementary figures and images for: Quantitative Assessment of Tumor Contact with Neurogenic Zones and Its Effects on Survival: Insights beyond Traditional Predictors
Source: Cancers (Basel). 2024 Apr 29;16(9):1743. doi: 10.3390/cancers16091743 (PMC11083354; doi:10.3390/cancers16091743)

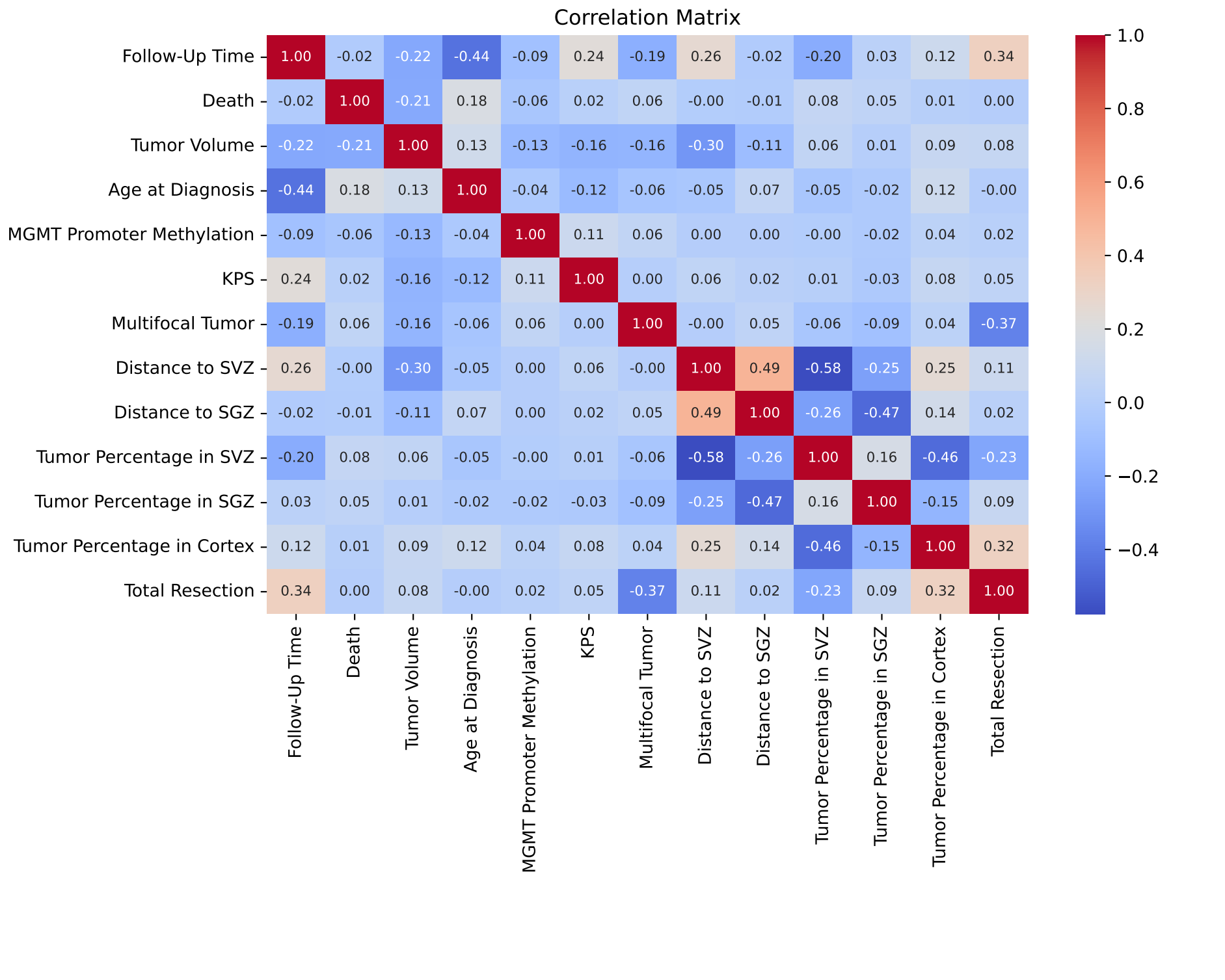

Supplement: Supplementary file 1 [file cancers-16-01743-s001.zip › cancers-2953692-supplementary.pdf]
